# Supplementary material for: Characterizing Intraindividual Podocyte Morphology In Vitro with Different Innovative Microscopic and Spectroscopic Techniques
Source: Cells. 2023 Apr 25;12(9):1245. doi: 10.3390/cells12091245 (PMC10177567; doi:10.3390/cells12091245)
Supplement: Supplementary file 1 [file cells-12-01245-s001.zip › cells-2334053-supplementary.pdf]

Supplementary Materials:

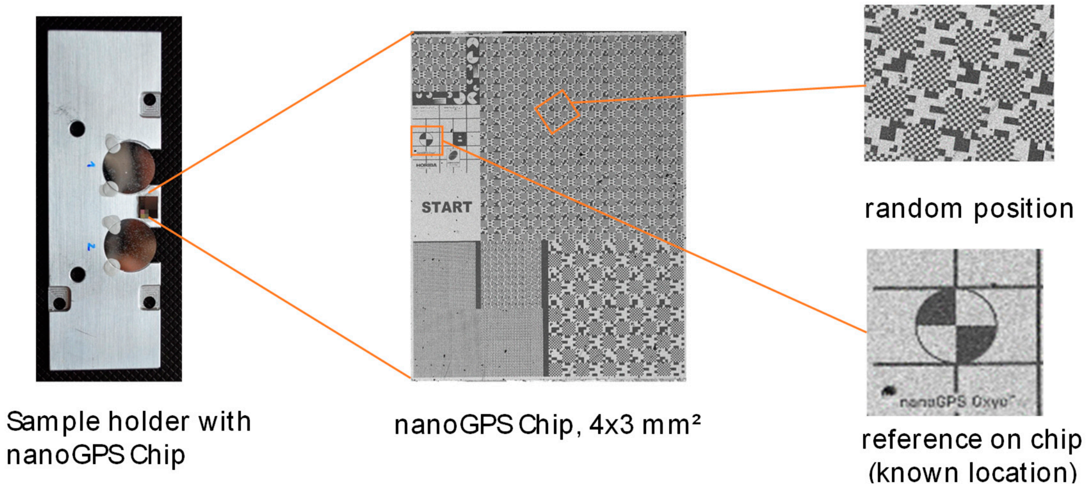

Supplementary Figure S1: Schematic workflow for nanoGPS Oxyo® tag calibration. Left: sample holder with nanoGPS Oxyo® tag and podocyte specimens; middle: pattern of the nanoGPS Oxyo® tag with different structures and sizes; top right: random position on the nanoGPS Oxyo® tag for calibration of the microscopes; bottom right: start-reference on the nanoGPS Oxyo® tag to cross-proof calibration
